# A news-based climate policy uncertainty index for China

**DOI:** 10.1038/s41597-023-02817-5

**Published:** 2023-12-08

**Authors:** Yan-Ran Ma, Zhenhua Liu, Dandan Ma, Pengxiang Zhai, Kun Guo, Dayong Zhang, Qiang Ji

**Affiliations:** 1 Institutes of Science and Development, Chinese Academy of Sciences, Beijing, China; 2 School of Public Policy and Management, University of Chinese Academy of Sciences, Beijing, China; 3 School of Economics and Management, China University of Mining and Technology, Xuzhou, China; 4 School of Economics and Management, Beihang University, Beijing, China; 5 School of Economics and Management, University of Chinese Academy of Sciences, Beijing, China; 6 Research Institute of Economics and Management, Southwestern University of Finance and Economics, Chengdu, China

**Keywords:** Climate-change mitigation, Governance

## Abstract

Climate policies can have a significant impact on the economy. However, these policies have often been associated with uncertainty. Quantitative assessment of the socioeconomic impact of climate policy uncertainty is equally or perhaps more important than looking at the policies themselves. Using a deep learning algorithm—the MacBERT model—this study constructed indices of Chinese climate policy uncertainty (CCPU) at the national, provincial and city levels for the first time. The CCPU indices are based on the text mining of news published by a set of major newspapers in China. A clear upward trend was found in the indices, demonstrating increasing policy uncertainties in China in addressing climate change. There is also evidence of clear regional heterogeneity in subnational indices. The CCPU dataset can provide a useful source of information for government actors, academics and investors in understanding the dynamics of climate policies in China. These indices can also be used to investigate the empirical relationship between climate policy uncertainty and other socioeconomic factors in China.

## Background & Summary

The *Global Risks Report 2023*^1^ released by the World Economic Forum indicates that the failure of climate action will be the dominating global risk factor in the next decade. To address climate change, countries worldwide have actively participated in global climate governance by formulating and implementing a series of related policies. However, climate uncertainty greatly affects climate policies’ execution and effectiveness^2^. The impact of climate policy uncertainty (CPU) is also a new source of risk that affects macroeconomic and financial systems. For example, the unexpected introduction of climate-related policies will trigger changes in investor preferences, increase uncertainties of market expectations and lead to an increase in the risk of stranding carbon-intensive assets, which poses a threat to financial stability^3^.

Given the continuous introduction of climate policies in various countries, it is crucial to quantify the uncertainty and time-varying characteristics of climate policies to help governments, businesses and investors make decisions. However, as CPU cannot be directly observed, quantifying its level is a key issue in assessing its impact and consequences. Existing studies have mainly measured CPU from three perspectives. One uses a single climate policy event to determine CPU^4^, which is problematic because this can only reflect the uncertainty caused by a single climate policy. The second uses non-economic dummy variables as proxy indicators to measure CPU. For example, Ilhan *et al*.^5^ identified the United States presidential election in 2016 as a policy shock to reflect short-term changes in CPU. The third involves the construction of a CPU index based on media information, such as that from newspapers^6^. Compared to the first two methods, the uncertainty index construction method based on news text information offers better traceability, time variation and sustainability and is being gradually applied to empirical research on the impacts of climate policy shocks on economic and financial risks. For example, Gavriilidis^6^ used data from eight major US newspapers to develop an uncertainty index for climate policy and examined the impact of CPU on carbon emissions. Similarly, Faccini *et al*.^7^ constructed CPU index for the United States based on news data and examined CPU’s macroeconomic impacts.

As the world’s largest emitter of greenhouse gases and second-largest economy, China bears important responsibilities in the course of climate change and has formulated and implemented a series of climate change-related policies^8^. China’s climate policies and their uncertainties have substantial implications for global climate governance. However, assessments of the uncertainty of China’s climate policy are still lacking. Only Lin and Zhao^9^ had constructed CPU indices for China, India, the United States and the United Kingdom. These indices have been used to examine the impact of CPU on economic activities. Xu *et al*.^10^ constructed the Chinese climate policy uncertainty (CCPU) index to determine the impact of CPU on the stock market. Overall, research gaps remain regarding the measurement of CCPU index. From the perspective of research methods, previous studies were based on the manual setting of rules to construct indices, in which text data categories were determined based on factors such as manually set keywords, and indices were then constructed based on the number of text categories. The major drawback of this method is its heavy reliance on rules and dictionary settings^9,10^, resulting in subjective selection bias in the research results. Second, it is difficult to handle ambiguous words in the text that are not in the dictionary, resulting in inaccurate text semantics and imprecise text classification. Finally, the used method in the previous literature has poor transferability and high requirements in terms of the quantity and quality of the training corpus^9,10^. If the corpus is not comprehensive and representative, the results and effectiveness of the classification model will be negatively impacted. Therefore, when using an index construction method based on rules and dictionaries, it is difficult to comprehensively and accurately mine CPU information in text.

From the perspective of data granularity, existing studies have mainly focused on CPU indices at the national level, whereas similar measures at the provincial and city levels are often needed, especially for China. Provinces in China significantly differ in climate condition, geographical location, energy structure, industrial structure and resource endowment, and provinces, cities and regions vary according to the key areas that must be targeted by climate policies^11,12^. In addition, China’s climate policy implementation features numerous regional pilot demonstrations and testing, creating a strong need for the construction of regional-level indices.

For the above-mentioned reasons, this study uses a deep learning algorithm, the MacBERT model, to develop the CCPU index for the national, provincial and city levels using data from January 2000 to December 2022. Unlike the rule- or dictionary-based textual analysis models or machine learning method^13–15^, the MacBERT model does not depend on an existing knowledge base or dictionary and thus avoids potential biases inherent in some existing works^6,9,10^. In addition, the flexibility of this model allows it to be generalisable to many scenarios^16–18^. Based on the deep learning of a large volume of Chinese texts, the MacBERT model can effectively extract general linguistic patterns and features. The model can automatically and accurately capture information from an entire sentence, generating distinct vector representations for each word in various contextual positions. Given these advantages, pre-trained language models, such as BERT and MacBERT, have gradually become the main tools for the text mining of policy and financial documents in recent years^19–21^. The foundation of these indices is based on 1,755,826 articles published by six mainstream Chinese newspapers: *People’s Daily*, *Guangming Daily*, *Economic Daily*, *Global Times*, *Science and Technology Daily* and *China News Service*. Manual auditing of the selected samples was done to assist in the deep learning process and check validity.

This study found that when the Chinese government introduced important regulatory policies and tools or changed regulatory attitudes, clear fluctuations occurred in the CCPU index. The main contributions of this article are as follows: First, from a technical perspective, the combination of manual auditing and a deep learning model based on textual analysis of news can avoid the subjectivity present in extant studies^6,9,10^, which have mainly used statistical keyword methods to construct CCPU indicators. The second advantage of this study relative to existing works^9,10^ is that it expands the national-level CPU index to the provincial and city levels. This work also expands the frequency of the CCPU index from monthly to daily, enriching its information content and providing critical sources for research in the high-frequency domain. The dataset can be useful to policymakers, practitioners and researchers who aim to evaluate the impacts of policy uncertainties and form optimal strategies for hedging against such uncertainties.

## Methods

**Fig. 1:**
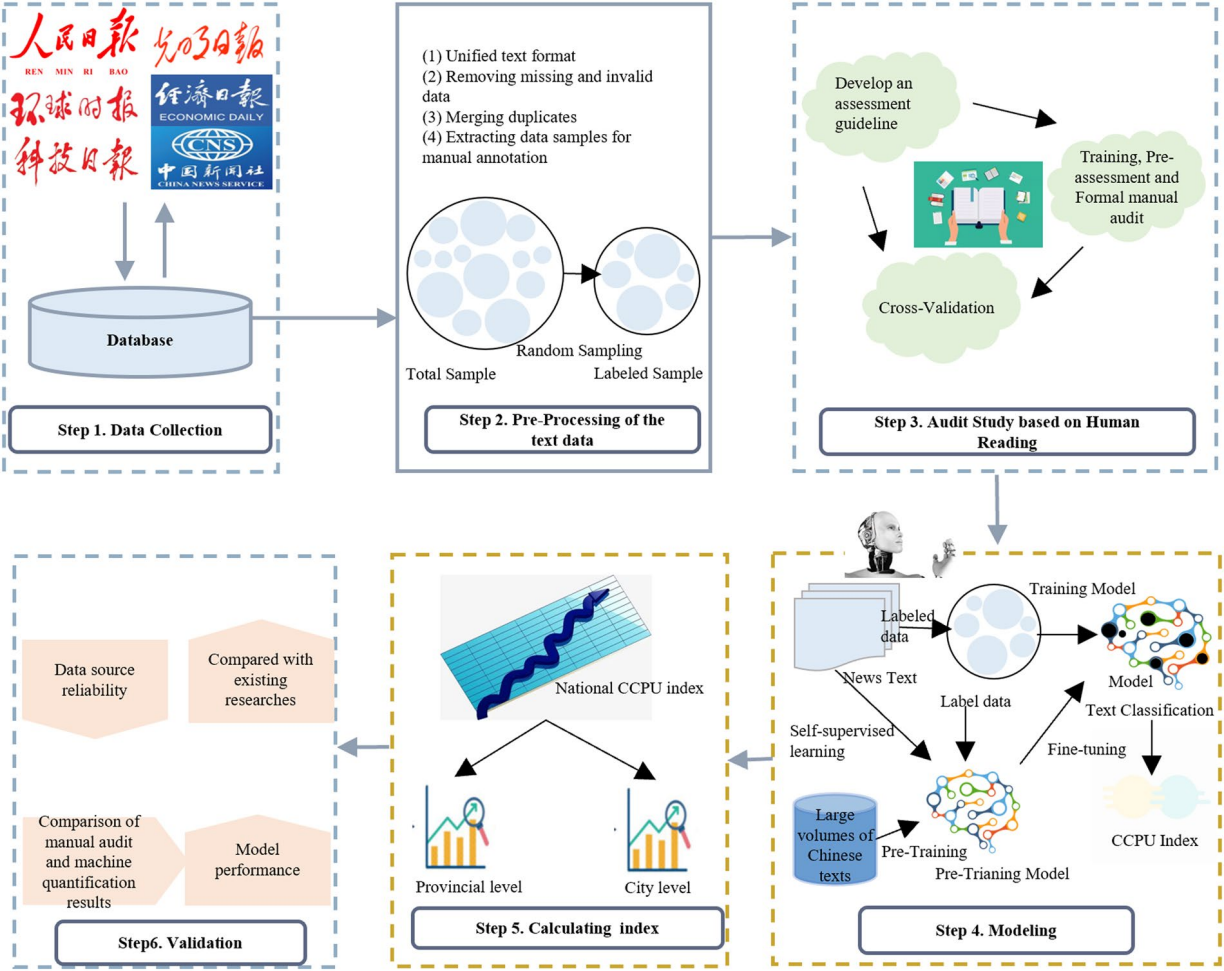
Research framework.

To construct the CCPU indices, mainstream newspapers in China were reviewed, and information related to both climate policies and uncertainties was extracted. This process consisted of six main steps: data collection, data cleaning, manual auditing, model construction, index calculation and validation. The general procedure is depicted in Fig. 1.

### Data collection

This work follows previous studies^22,23^ in choosing the source of information. The following criteria are used: **(1) Credibility**. News media outlets are selected based on their position in the popularity hierarchy and public trustworthiness by referencing the *List of Internet Information Source Units*, which was released by the Cyberspace Administration of China. **(2) Influence**. The impact of policy dissemination by newspapers is also considered. For this information, the *Top 100 List of Newspaper Convergence Communication* released by the People’s Daily Online Research Institute in 2020, which highlights the significant role of newspapers in shaping public opinion and policy discourse, is consulted. **(3) Internationalisation**. Climate change is an international issue; thus, CCPU should also reflect uncertainties in the international environment, and the selected news media should feature international perspectives. This is reflected in whether the newspaper has an English version or versions in other languages. Overall, six newspapers are chosen as the main sources for constructing the CCPU index: *People’s Daily*, *Guangming Daily*, *Economic Daily*, *Global Times*, *Science and Technology Daily* and *China News Service*. The newspaper data are collected from the WiseNews database between January 2000 and December 2022. The indices can be updated regularly on a monthly basis.

### Data cleaning

Given the inconsistencies in news formats and structures, which vary from a few sentences to several paragraphs, article titles and the main text are first combined into a single text document. Further text cleaning is performed to ensure data quality and to improve the accuracy and reliability of the model. This cleaning process primarily involved the removal of extraneous information that is least relevant to the research, such as punctuation, URLs and spaces.

### Manual auditing

CCPU refers to the uncertainty associated with various aspects of climate policies, including the entities responsible for policy formulation (*who develops climate policies*), the issuance timing and policy content (*when and what types of policies are implemented*) and the consequences of the implementation of climate policies (*outcomes of climate policy actions*). A manual auditing team comprising master’s and doctoral students in the field of economics and finance is first assembled to determine whether a news item contains uncertainty on climate policy. Each member is asked to assess whether a news item contained a CCPU by manually reading the whole context of the article. The news item was then labelled CPU = 1 if yes, indicating that the news item contained CPU. Each item is assessed independently by multiple readers to ensure reliability. The detailed steps are as follows:

1. **Form an auditing guide**. Each auditing team member reads news related to climate policy and invests two months in this endeavour. During this process, team members collectively formulate a standardised approach to recording the results of the manual audits. A manual assessment guide is also developed to ensure consistency and accuracy in the assessment process. The guideline provides detailed explanations of the auditing rules, an assessment template, frequently asked questions and several examples of auditing studies. These resources are provided to help the auditing team better understand the requirements for completing manual auditing accurately.
2. **Training and pre-assessment**. Next, an auditing team consisting of 48 master’s and doctoral students specialising in economics or finance from universities such as the University of Chinese Academy of Sciences, Southwestern University of Finance and Economics, China University of Mining and Technology and the University of Science and Technology of Macau is created. Relevant training is provided to the auditing team members, and each is assigned 100 news articles to rate. Based on the preliminary assessment results, further training is provided to the team members, and the guidelines undergo continued revision and refinement. Following several iterations, a pre-assessment result accuracy rate higher than 96% is achieved.
3. **Formal auditing**. Each team member is assigned 800 news items to evaluate. The assessment results are then used for subsequent training and evaluation of the deep learning models. To increase the efficiency of the formal process, news items that have already been read are removed, and then a total of 28,800 news items are randomly and proportionally selected as the reading sample. Forty-eight auditors are assigned to 16 teams to make sure that each news item would be read by three auditors independently. During the formal manual auditing phase, group discussions are scheduled to recapitulate the challenges faced during the auditing process and continually improve the guidelines. A total of 4 months are required for all auditors to finish reading and rating the news samples.
4. **Cross-validation**. News items with inconsistent results among the three auditors in the auditing teams are reassigned to another group of auditors for additional assessment. The new results are returned to the original team for feedback and to ensure consistency. In addition, the research team spent 1 month examining the manual auditing results.

### Learning with the MacBERT model

**Fig. 2:**
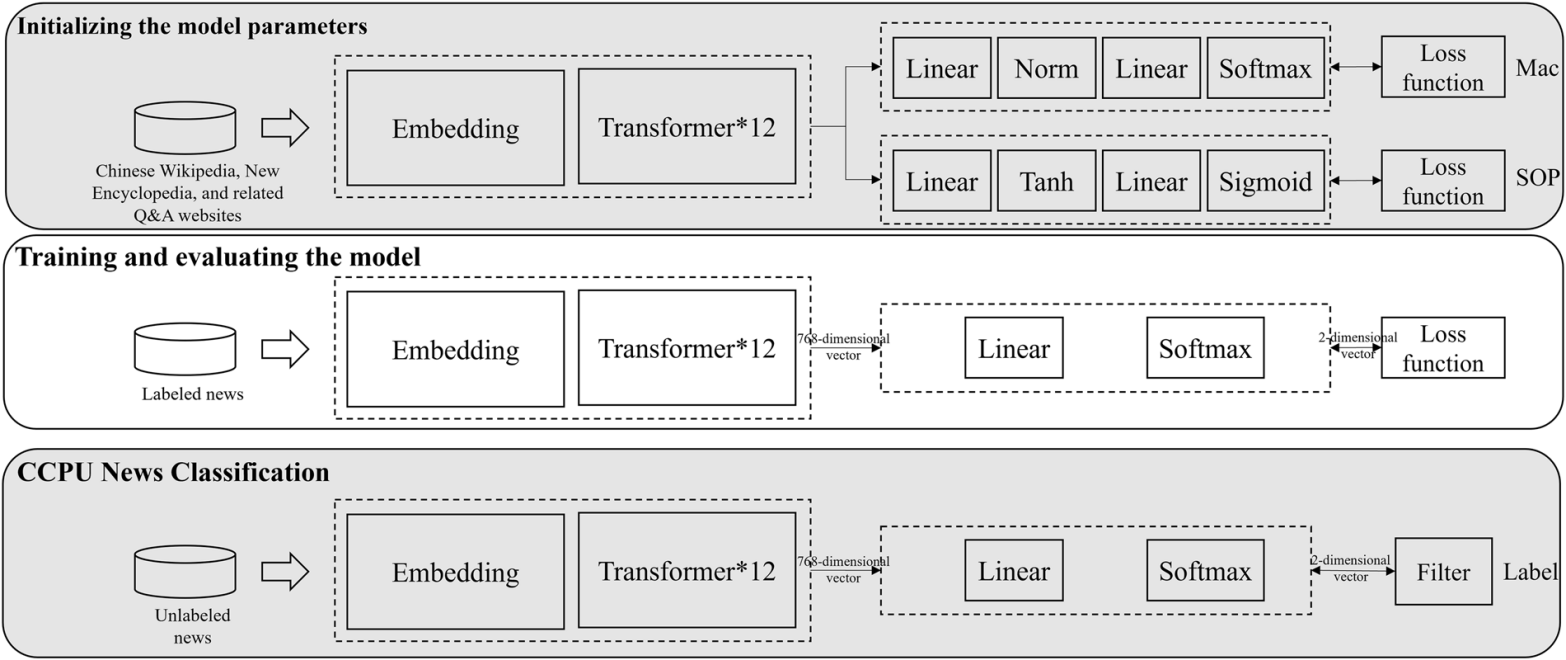
Model algorithm process.

The MacBERT^24^ deep learning model is used to evaluate the news and construct the CCPU index. In general, the modelling process consists of three steps (see Fig. 2).

1. **Initialising the model parameters**. Large volumes of Chinese texts (e.g. Chinese *Wikipedia*, New Encyclopedia and related Q&A websites) are used to train the model to learn Chinese language rules and features. At the same time, the model automatically obtains vector representations of the target texts. To enhance the pre-trained language model’s acquisition of knowledge, this research avoids training the base-level model from scratch; instead, the initial parameters of the official MacBERT-Base-Chinese are adopted^24^.
2. **Training and assessment**. Here, the labelled datasets from manual auditing are used to train the MacBERT parameters to better reflect the relevance of CCPU-related news. In this stage, 70% of the labelled datasets are randomly selected as the training datasets, and 30% of the data are left as testing datasets. During the training phase, classification functionality is realised through the linear layer, which generate scores relative to each class label. The Softmax activation function then converted these scores into probabilities. Standard cross-entropy loss is used to optimise the training task.
  1 $$L\left(\overrightarrow{y},\overrightarrow{p}\right)=-\mathop{\sum }\limits_{i=1}^{2}{y}_{i}\;\log \;{p}_{i}$$
  $L\left(\overrightarrow{y},\overrightarrow{p}\right)$ is the classification loss value for each news item, $\overrightarrow{y}$ is a two-dimensional vector and represents the actual classification of the news and the *i*-th element ${y}_{i}$ in $\overrightarrow{y}$ indicates whether the news belongs to the *i*-th classification of labels. If ${y}_{i}=1$, the news belongs to the *i*-th classification; otherwise, ${y}_{i}=0$. $\overrightarrow{p}$ is also a two-dimensional vector and represents the output of the model, and the *i*-th element ${p}_{i}$ in $\overrightarrow{p}$ indicates the probability that the news belongs to the *i*-th class label.
3. **CCPU news classification**. After training and assessment, the model is used to classify unlabelled news directly. Compared to the architecture of the trained model, the only noticeable difference is the addition of the filter after the Softmax activation function. Softmax outputs the probability ($\overrightarrow{p}$) that the claimed text belongs to various types of labels, and the filter outputs the labels with the largest probability. Using the algorithm outlined above, more than 1.75 million news items from six distinct newspapers spanning 2000–2022 are classified.

### Calculating the CCPU index

After classifying the CPU articles and accounting for the number of these articles in a particular period, raw CCPU data are obtained. The data are then standardised to construct the CCPU index. The principle of Baker *et al*.^22^ is followed to calculate and standardise the CCPU index. This approach effectively eliminates bias caused by variations in the number of news reports published by newspapers in different periods.

Specifically, let *X*_*it*_ denote the ratio of the number of CCPU news items in month *t* of the *i-*th newspaper to the number of news items issued in the same month. To aggregate over newspapers and construct the monthly CCPU index, the following steps are taken: (1) Compute the time-series variance, *σ*_*i*_, in the interval *T* for each newspaper *i*; (2) Standardise *X*_*it*_ by dividing through by the standard deviation *σ*_*i*_ for *t*—the operation yields for each news a series *Y*_*it*_ with a unit standard deviation in the interval *T*; and (3) Compute the average over newspapers of *Y*_*it*_ in each month *t* to obtain the series *Z*_*t*_, which is the normalised CCPU index.

### Validation

As the parameters in the model are primarily determined by the training set, changes in the samples can affect the value of the parameters and potentially result in errors. To address this problem, three rounds of random sampling and cross-validations are conducted in the manual auditing process to identify optimal parameters. In addition, to ensure that the constructed indices are reliable, the method described in the existing literature is adhered to in performing a set of cross-validations. The source data are based on six major newspapers. To address the concerns about the data sources, news outlets, such as specialised newspapers, is added as the foundation in constructing indices. Some typical model performance criteria are constructed based on the criteria previously described in the literature to check the superiority of the proposed deep learning model. The results are compared to the manual auditing results and alternative deep learning models to ensure that the MacBERT model works properly. This study acknowledges that previous studies have attempted to measure CCPU. Despite the limitations of existing works, prior results are used for comparison with this study’s dataset and the validation of the indices.

## Data Records

The CCPU dataset is publicly available on Figshare. There are four items in the repository. The files contained in the dataset are listed in Table 1.

- **China’s CPU index:** This item contains the national CPU dataset in China from 2000 to 2022, including daily, monthly and annual indices. An Excel data file is provided in Figshare^25^.
- **China’s provincial CPU index:** This item contains CPU dataset of 31 provinces in China from 2000 to 2022 (excluding data from Hong Kong, Macao and Taiwan), including monthly and annual indices. An Excel data file is provided in Figshare^26^.
- **China’s city-level CPU index:** This item contains CPU dataset of 293 cities in China from 2000 to 2022 (excluding Hong Kong, Macao and Taiwan), including monthly and annual data. An Excel data file is provided in Figshare^27^.
- **Dataset of newspaper:** Data from the six newspapers chosen as the main sources for constructing the CCPU index (i.e. *People’s Daily*, *Guangming Daily*, *Economic Daily*, *Global Times*, *Science and Technology Daily* and *China News Service*) are provided. The newspaper data featuring items published between January 2000 and December 2022 are collected from the WiseNews database. The dataset of 1,755,826 articles is stored in Figshare^28^.

**Table 1:** Summary of the dataset.

Level	Content	Document name	Sheet name
**1. National level**	National CPU dataset in China	China’s CPU index	Daily
			Monthly
			Annual
**2. Province level**	CPU dataset of 31 provinces in China	China’s provincial-level CPU index	Monthly
			Annual
**3. City level**	CPU dataset of 293 cities in China	China’s city-level CPU index	Monthly
			Annual
**4. Other information**	Data of original newspapers	Dataset of newspaper items	/

## Technical Validation

### Including more newspapers

Climate change policies are closely linked to energy use and the environment^29^. Therefore, a combined approach using authoritative general newspapers in conjunction with specialised newspapers focusing on energy and the environment is employed. Specifically, *China Energy News*, *China Science Daily*, *XinHua Daily Telegraph* and *China Environment News* are chosen as the supplemental data sources for the recalculation of the alternative CCPU index. A correlation analysis is performed between the alternative and previously constructed CCPU index. The correlation coefficient between the two indices is 84.47%, indicating that the main CCPU index is robust to the selection of news sources.

### Comparison with manual auditing

Four indicators, specifically accuracy, precision, recall and F1 score (F1), are chosen for use in assessing the degree of consistency between the two results (manual auditing and deep learning). Accuracy is calculated as the percentage of total samples with correct prediction results. Precision is the probability of actual positive samples among all the samples predicted to be positive. Recall is the probability of being correctly predicted. F1 is a combination of precision and recall. The specific formula used is as follows:

2 $$Accuracy=\frac{TP+TN}{TP+FP+FN+TN}$$

3 $$Precision=\frac{TP}{TP+FP}$$

4 $${Recall}=\frac{TP}{TP+FN}$$

5 $$F{\rm{1}}=\frac{2\times Precision\times Recall}{Precision+Recall}$$

News with CPU is defined as positive (P) news, and news items without CPU are defined as negative (N) news. The deep learning procedure is used to judge (predict) whether the news is positive or negative. TP denotes the number of correct predictions of positive news (true positive), and FP is the number of incorrect predictions of positive news (false positive). Similarly, TN refers to the number of correct predictions of negative news (true negative), and FN refers to the number of incorrect predictions of negative news (false negative). Therefore, accuracy is determined based on all predictions of positive news and negative news, precision only depends on news with positive predictions (true + false) and recall denotes the probability of a positive prediction being true.

The accuracy, precision, recall and F1 results of the MacBERT model are 97.83%, 97.96%, 98.85% and 98.40%, respectively, indicating a high degree of consistency between the quantitative results of the MacBERT model and the audit study results. In addition, Cohen’s Kappa and intraclass correlation coefficient (ICC) tests are conducted to assess the validity and reliability of the manual auditing process. The Cohen’s Kappa is 0.843, and the ICC score is 0.843/0.915 (single/average), indicating a high level of consistency.

### Model performance

To ensure that the results are not due to model selection and to check the robustness of the results, alternative models are used, and the new results are compared with those from the MacBERT model. The Lert, Pert and Roberta models are selected as alternative models. The same dataset (30% of the dataset in the labelled set that are randomly selected as the testing dataset) is used to evaluate the model’s performance. The results presented in Table 2 show that among all models, the MacBERT model has the highest accuracy and F1 score, is second in precision and third in recall among all four models. In other words, the overall performance of the MacBERT model is optimal.

**Table 2:** Performance comparison results of the four models on the testing dataset.

	MacBERT	Lert	Pert	Roberta
**Accuracy**	93.33%	93.27%	92.87%	93.04%
**Precision**	93.73%	93.00%	94.03%	91.94%
**Recall**	96.51%	96.98%	95.92%	96.62%
**F1**	95.10%	94.95%	94.96%	94.22%

The CCPU index is then calculated using the three new models. The new indices are compared to the CCPU index resulting from the MacBERT model. The correlations are 98.2%, 98.4% and 98.6% for the Lert, Pert and Roberta models, respectively. In general, the results are not affected by the model selection and are thus considered robust.

### Comparison with existing studies

Before this study, few studies have attempted to empirically evaluate CPU in China (e.g. Xu *et al*.^10^ and Lin and Zhao^9^). This is the first news-based deep learning study. To evaluate the superiority of the proposed index construction method, the CCPU index is constructed using the testing dataset, following the methods of Xu *et al*.^10^ and Lin and Zhao^9^. The model performance results are shown in Table 3. As seen from the results, all indicators demonstrate that the proposed approach using the MacBERT model performs better. It is worth noting that the MacBERT model requires a relatively higher time cost for pre-training and demands specific computational resources. These costs are, however, within an acceptable range; therefore, this powerful model can be extended to other textual analyses.

**Table 3:** Model performance of the MacBERT model versus existing works.

	MacBERT	Xu *et al*.	Lin and Zhao
**Accuracy**	93.33%	37.69%	34.20%
**Precision**	93.73%	81.98%	83.76%
**Recall**	96.51%	10.44%	3.72%
**F1**	95.10%	18.52%	7.12%

### Limitations and future work

Understanding the impacts of CCPU is often more important than that of individual climate policies. The aim of this paper is to fill this gap by constructing a robust CCPU measure for China. Specifically, manual auditing and deep learning models are combined to construct CCPU indices based on news text. Although the constructed indices are demonstrated to be robust and can depict the dynamics in China, limitations remain, such as the lack of indices classified by topic.

First, the indices are constructed using all climate policies. In other words, this work does not distinguish between different types of climate policies. For example, economic- and finance-related policies may be separated, as the implications of these sub-categories differ. For example, financial investors may be more interested in finance-related policy uncertainties. Second, although this study demonstrates the need to highlight regional differences in terms of CCPU at the provincial and city levels, the indices constructed can be improved through the adoption of localised news outlets. Doing so will affect the general comparability of these local indices.

Considering these potential extensions, the researchers plan to further investigate this issue by disaggregating CPU further and creating specific sub-indices to facilitate research/investment needs by different types of audiences. Moreover, given the robustness and reliability of the deep learning model, it is easy to expand the study targets to other areas, including the use of more detailed and localised news information.

### Supplementary information

Supplementary information

## Data Availability

The codes that were used to generate the CCPU dataset are available in Figshare^30^.
