# Supplementary material for: A news-based climate policy uncertainty index for China
Source: Sci Data. 2023 Dec 8;10:881. doi: 10.1038/s41597-023-02817-5 (PMC10709629; doi:10.1038/s41597-023-02817-5)
Supplement: Supplementary file 1 — Supplementary information [file 41597_2023_2817_MOESM1_ESM.docx]

**Supplementary Materials**

**Table of contents**

[Supplementary 1: CCPU index at the national, provincial and city levels 2](#_Toc151921578)

[Supplementary 2: Comparison between the CCPU index and the US CPU index 6](#_Toc151921579)

[Supplementary 3: Computational resources for the MacBERT model 7](#_Toc151921580)

# Supplementary 1: CCPU index at the national, provincial and city levels

**CCPU at the national level.** As shown in Fig. S1, China’s national-level CPU index shows a general upward trend, indicating stronger policy uncertainties in the country. The CCPU index increased significantly during periods when important climate actions are taken. For example, around the signing of the ‘Copenhagen Agreement’ in December 2009, the CCPU index reflected a sharp increase, indicating general uncertainties about what actions will be taken by the Chinese government. A similar case occurred when China announced its dual-carbon target in September 2020 for peak CO_2_ emissions to occur by 2030 before working towards carbon neutrality by 2060^1^. The Chinese government released a series of documents, including *Working Guidance for Carbon Dioxide Peaking and Carbon Neutrality in Full and Faithful Implementation of the New Development Philosophy*^2^. Since then, the level of uncertainty in climate policy has increased sharply to a historical high.

**
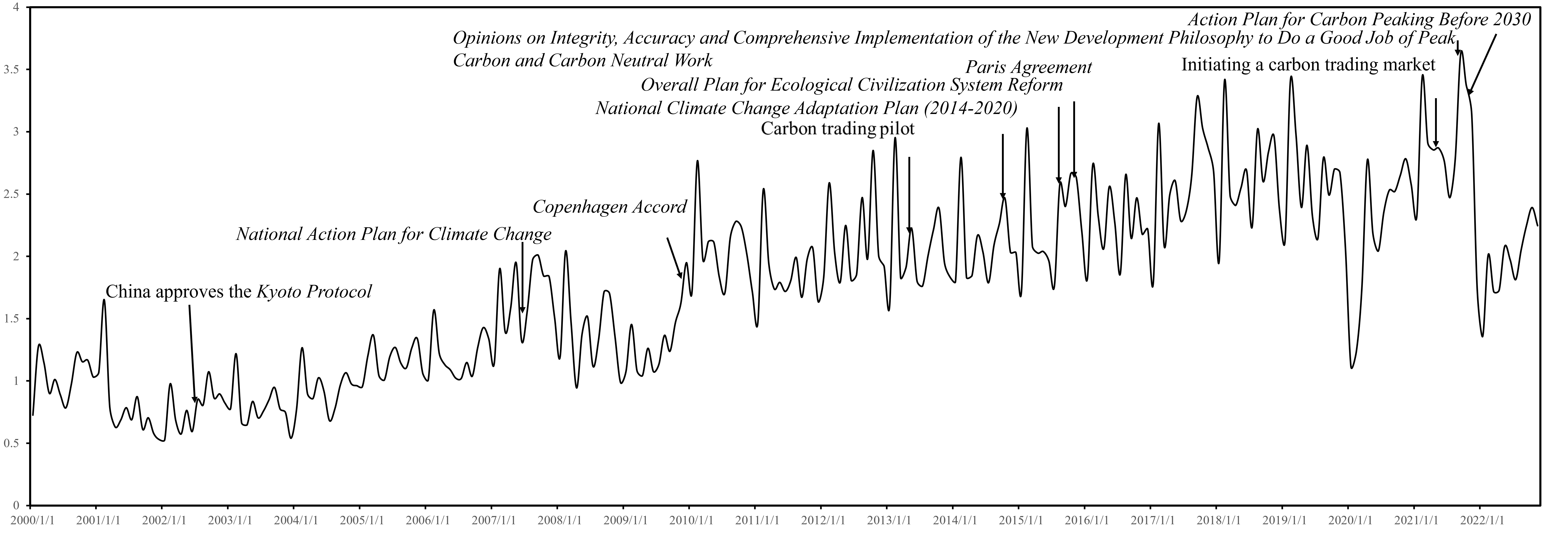
**

**Fig. S1. CCPU index at the national level.**

According to the graphical pattern, the CCPU index can be roughly divided into three stages. The first stage occurred from 2000 to 2009, during which the CCPU index was generally low. The second stage was from 2009 to 2015, during which the CCPU index gradually increased. The third stage is from 2016 to the present or since the signing of the Paris Agreement, during which the CCPU index has shown a high rate of fluctuation. These stages are generally consistent with expectations as to when climate change began attracting increasing attention in the country. At the same time, they reveal the challenges caused by climate change. Policymakers must balance the demand for economic development and long-term sustainability, which leads to increased uncertainty.

**CCPU index at the provincial level.** Fig. S2 plots the CCPU index of 31 provinces from 2000 to 2022. The overall trend at the provincial level is similar to that at the national level. The index in most provinces showed a clear upward trend and experienced sharp increases around the time of major climate actions. For example, in June 2007, the State Council established the National Leading Group for Climate Change Response and Energy Conservation and Emission Reduction and immediately released the ‘National Plan for Climate Change Response’^3^. In July 2008, the National Development and Reform Commission established the Climate Change Response Department to be responsible for specific work, such as policy formulation, international negotiations, capacity building and carbon market construction. Several provinces and cities have established specialised functional agencies for the Climate Change Response Department. In September 2021, the Central Committee of the Communist Party of China and the State Council issued the *Working Guidance for Carbon Dioxide Peaking and Carbon Neutrality in Full and Faithful Implementation of the New Development Philosophy*^2^, and 31 provinces, municipalities and autonomous regions successively issued action plans to implement carbon peak and neutrality goals^4^. There are also clear heterogeneities across provinces. For example, the CCPU indices for the northern regions, such as the provinces of Jilin and Liaoning, are clearly flatter than others and deserve further investigation.


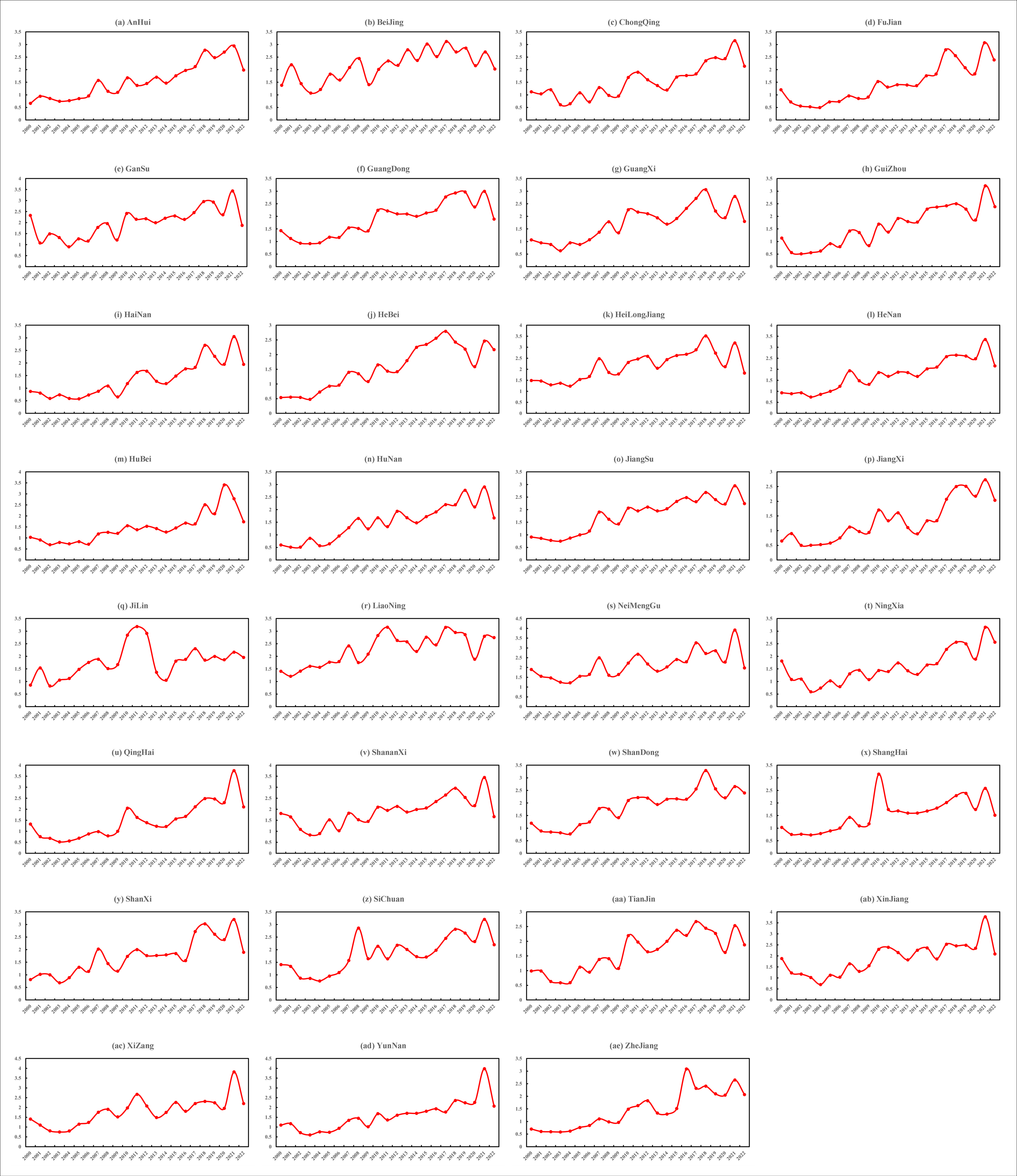


**Fig. S2. CCPU index at the provincial level.**

**CCPU index at the city level.** CCPU indices at the prefectural city level were constructed. These city-level indices also showed clear heterogeneity, mainly including three typical trends. Fig. S3 plots the CCPU indices for three typical cities in China from 2000 to 2022, each with two examples. Hefei and Nanjing are in the first category, with most cities in China sharing the same upward trend, which is similar to that of the national and provincial levels. The cities of Wenzhou and Changchun are examples of the second group, which experienced a significant increase in uncertainties around 2010–2012. These types of cities showed no clear upward trend, and the indices are clearly flatter except for the sharp peak in the middle of the whole sample period. The last category of cities, such as Lishui and Huzhou, had a rather flat trend until 2014, followed by a steeper upward trend.


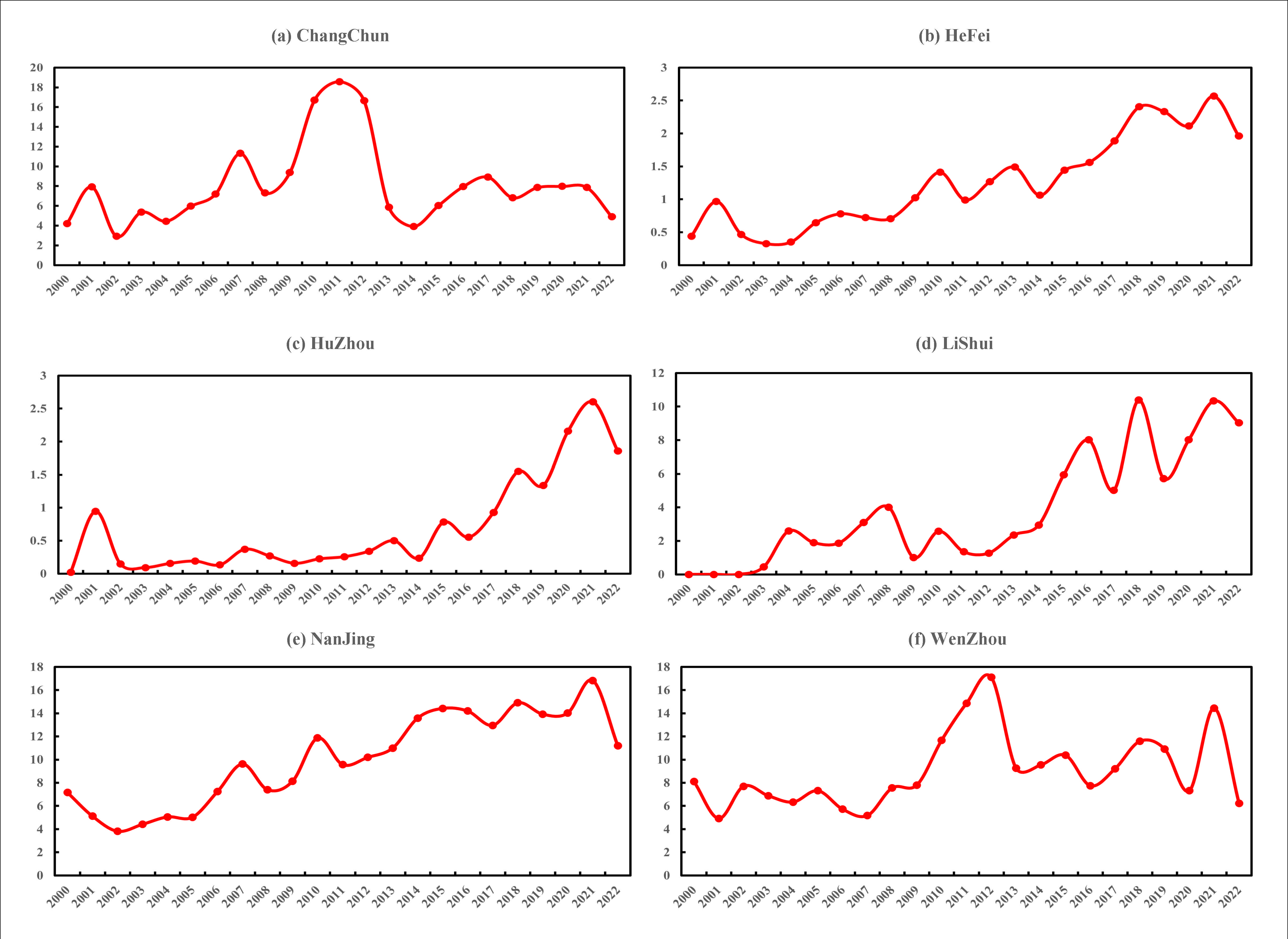


**Fig. S3. CCPU index at the city level.**

# Supplementary 2: Comparison between the CCPU index and the US CPU index

Here, the CCPU index is compared to the US CPU index^5^ in Fig. S4. There are clear similarities but also significant differences between them. These two indices share an upward trend over the entire sample period, reflecting the generally rising climate concerns and policies in both countries. CCPU began to rise after the 2008 global financial crisis, and a significant gap between these two indices existed until 2016, when US CPU started to rise and to match that in China. This is consistent with the intensive policy interventions in China to protect the environment and pursue green development.


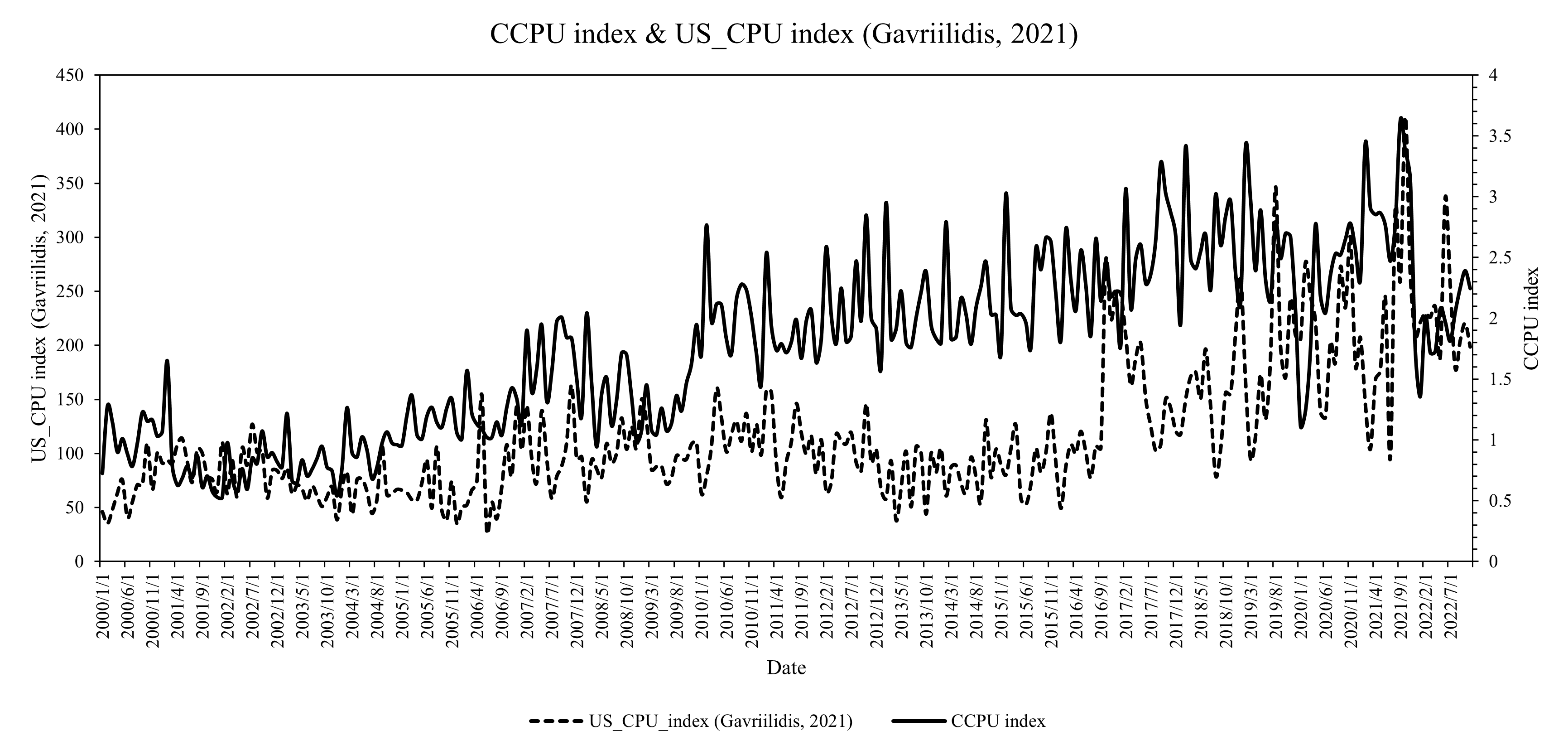


**Fig. S4. Comparison of the CCPU index and the US CPU index.**

# Supplementary 3: Computational resources for the MacBERT model

Computational resources for the MacBERT model in this paper are shown in Table S1.

**Table S1.** **Computational resources used to implement the MacBERT model**

| **Name** | **Information** |
| --- | --- |
| CPU | Intel Xeon e5-2680v3 |
| GPU | NVIDIA Geforce RTX 2080 Ti/ 11G |
| Memory | 32G ddr4 |
| OS | Windows 10 Professional |
| Software | Python 3.9; Cuda 11.7 |
| Package | Tourch 2.0; tqdm; rich; numpy; pycocotools; pandas; transformers; random; time; cv2 |

**References**

1. Zhang, G. *et al*. China’s environmental policy intensity for 1978–2019. *Sci. Data* **9,** 75 (2022).

2. The Communist Party of China Central Committee and the State Council. *Working Guidance For Carbon Dioxide Peaking And Carbon Neutrality In Full And Faithful Implementation Of The New Development Philosophy*. <https://english.www.gov.cn/policies/latestreleases/202110/25/content_WS61760047c6d0df57f98e3c21.html> (2021)

3. Wang, B., Hong, G., Cui, C. Q., Yu, H., & Murty, T. Comprehensive analysis on China’s National Climate Change Assessment Reports: Action and emphasis. *Front. Eng. Manag.* **6,** 52-61 (2019).

4. Liu, Z. *et al*. Challenges and opportunities for carbon neutrality in China. *Nat. Rev. Earth Environ.* **3,** 141-155 (2022).

5. Gavriilidis, K. Measuring climate policy uncertainty. Preprint at http://dx.doi.org/10.2139/ssrn.3847388 (2021)
